# Genome-wide association study of important agronomic traits within a core collection of rice (*Oryza sativa* L.)

**DOI:** 10.1186/s12870-019-1842-7

**Published:** 2019-06-17

**Authors:** Peng Zhang, Kaizhen Zhong, Zhengzheng Zhong, Hanhua Tong

**Affiliations:** State Key Laboratory of Rice Biology, China National Rice Research Institute, Hangzhou, 310006 China

**Keywords:** Ting’s core collection, Rice (*Oryza sativa* L.), Agronomic traits, Genome-wide association study, Elite genes

## Abstract

**Background:**

Cultivated rice (*Oryza sativa* L.) is one of the staple food for over half of the world’s population. Thus, improvement of cultivated rice is important for the development of the world. It has been shown that abundant elite genes exist in rice landraces in previous studies.

**Results:**

A genome-wide association study (GWAS) performed with EMMAX for 12 agronomic traits measured in both Guangzhou and Hangzhou was carried out using 150 accessions of Ting’s core collection selected based on 48 phenotypic traits from 2262 accessions of Ting’s collection, the GWAS included more than 3.8 million SNPs. Within Ting’s core collection, which has a simple population structure, low relatedness, and rapid linkage disequilibrium (LD) decay, we found 32 peaks located closely to previously cloned genes such as *Hd1*, *SD1*, *Ghd7*, *GW8*, and *GL7* or mapped QTL, and these loci might be natural variations in the cloned genes or QTL which influence potentially agronomic traits. Furthermore, we also detected 32 regions where new genes might be located, and some peaks of these new candidate genes such as the signal on chromosome 11 for heading days were even higher than that of *Hd1*. Detailed annotation of these significant loci were shown in this study. Moreover, according to the estimated LD decay distance of 100 to 350 kb on the 12 chromosomes in this study, we found 13 identical significant regions in the two locations.

**Conclusions:**

This research provided important information for further mining these elite genes within Ting’s core collection and using them for rice breeding.

**Electronic supplementary material:**

The online version of this article (10.1186/s12870-019-1842-7) contains supplementary material, which is available to authorized users.

## Background

Cultivated rice (*Oryza sativa* L.) is one of the staple foods for over half of the world’s population. Uncovering the genetic basis of natural variations in important agronomic traits in rice landraces is indispensable for ensuring the world’s food supply.

In general, linkage mapping is a conventional method for gene mining in rice. However, association mapping based on linkage disequilibrium (LD) has been widely used in rice studies since it was firstly reported in maize [1, 2]. Association mapping could overcome the limitations (i.e., limited alleles, high cost and poor mapping resolution) of linkage mapping [3] and enable researchers to use modern genetic technologies for exploiting natural genetic diversity and identifying elite genes in the genome [4]. Furthermore, many candidate genes or loci have been identified in rice through genome-wide association study (GWASs) of agronomic traits [5–10], abiotic stress tolerance [11–13] and metabolites [14, 15].

A population with diverse landraces or cultivars which could be used in crops GWASs is supposed to be a permanent resource and be rephenotyped for many traits [2]. Sampling populations (e.g., core collections and mini core collections) created from rice landraces might be a suitable choice for rice GWASs [16]. Rice landraces are easier to be utilized for breeding than wild rice because they have greater genetic diversity than elite cultivars and represent an intermediate stage of domestication history between wild rice and cultivars [17]. As early as 1920–1964, Ying Ting collected more than 7128 rice landraces from all over China and from some of the other main rice-cultivating countries. This collection is one of the earliest collections of rice germplasm resources in China and was named Ting’s collection [18]. Moreover, a rice core collection called Ting’s core collection and consisting of 150 accessions selected based on 48 phenotypic traits has been constructed from 2262 accessions of Ting’s collection [18]. In Ting’s core collection, the average polymorphism information content (PIC) is 0.48, and the average genetic diversity is 0.54 [19]. Furthermore, Ting’s core collection has been used in association mapping of 12 agronomic traits [20] and aluminum tolerance [21] with 274 SSR markers. However, no association mapping with higher resolution has been performed for agronomic traits within Ting’s core collection.

In the present study, a GWAS of 12 rice agronomic traits was carried out using Ting’s core collection of rice landraces with more than 3.8 million high-quality 3.8 million SNPs by whole-genome re-sequencing. Regions identified by the GWAS were compared with those identified as QTL and candidate genes in previous studies. This information will be very useful for rice breeders to improve elite cultivars.

## Results

### Comparison between Ting’s core collection and other populations used in GWASs

**Fig. 1:**
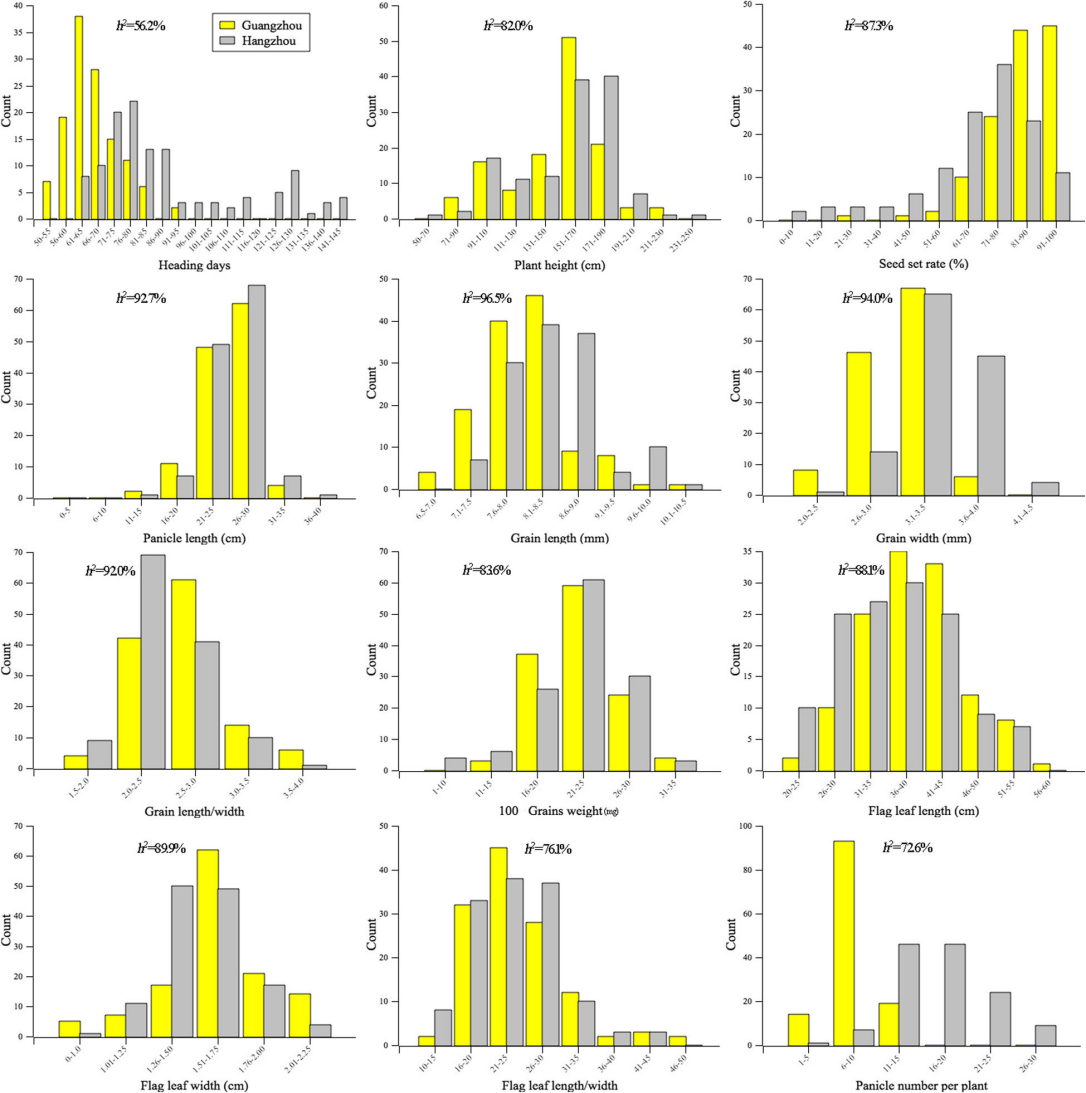
Frequency distribution of agronomic traits in Ting’s core collection

Ting’s core collection consists of 150 rice landraces that were collected from 20 different provinces of China and from North Korea, Japan, the Philippines, Brazil, Sulawesi, Java, Oceania, and Vietnam (Additional file 2: Table S1). The number of varieties in Ting’s core collection is lower than that in a population of Chinese rice landraces [5], a global collection [9] and a mini core collection of *japonica* rice [8], however, the phenotypic diversity in several agronomic traits in Ting’s core collection are comparable to those in above mentioned collections or even higher for some agronomic traits (Fig. 1).

### Genome re-sequencing and SNP identification

Whole-genome re-sequencing of Ting’s core collection was performed, resulting in a total of 522.4 Gb of clean data with an average sequencing depth of 7.3× and an average coverage of 82.9% of the reference genome (Additional file 2: Table S2). The distribution of SNP positions along each chromosome are shown in Additional file 1: Figure S1. A total of 3,808,730 SNPs and 391,756 InDels with a minor allele frequency > 0.05 were generated, and 386,562 SNPs were found in the CDS region (Additional file 2: Table S3).

### Phenotypic variation

A wide range of phenotypic variation in the 12 agronomic traits was revealed in Ting’s core collection both in Guangzhou and Hangzhou (Fig. 1). Plant height, grain length, grain width, grain length/width, 100 grains weight, flag leaf length, flag leaf width and flag leaf length/width showed similar distributions in the two locations, while heading days, seed set rate, panicle length and panicle number per plant had different distributions in the two locations. The broad-sense heritability ranged from 56.2% (Heading days) to 96.5% (Grain length) for these traits (Fig. 1).

### Population structure and LD estimation in Ting’s core collection

**Fig. 2:**
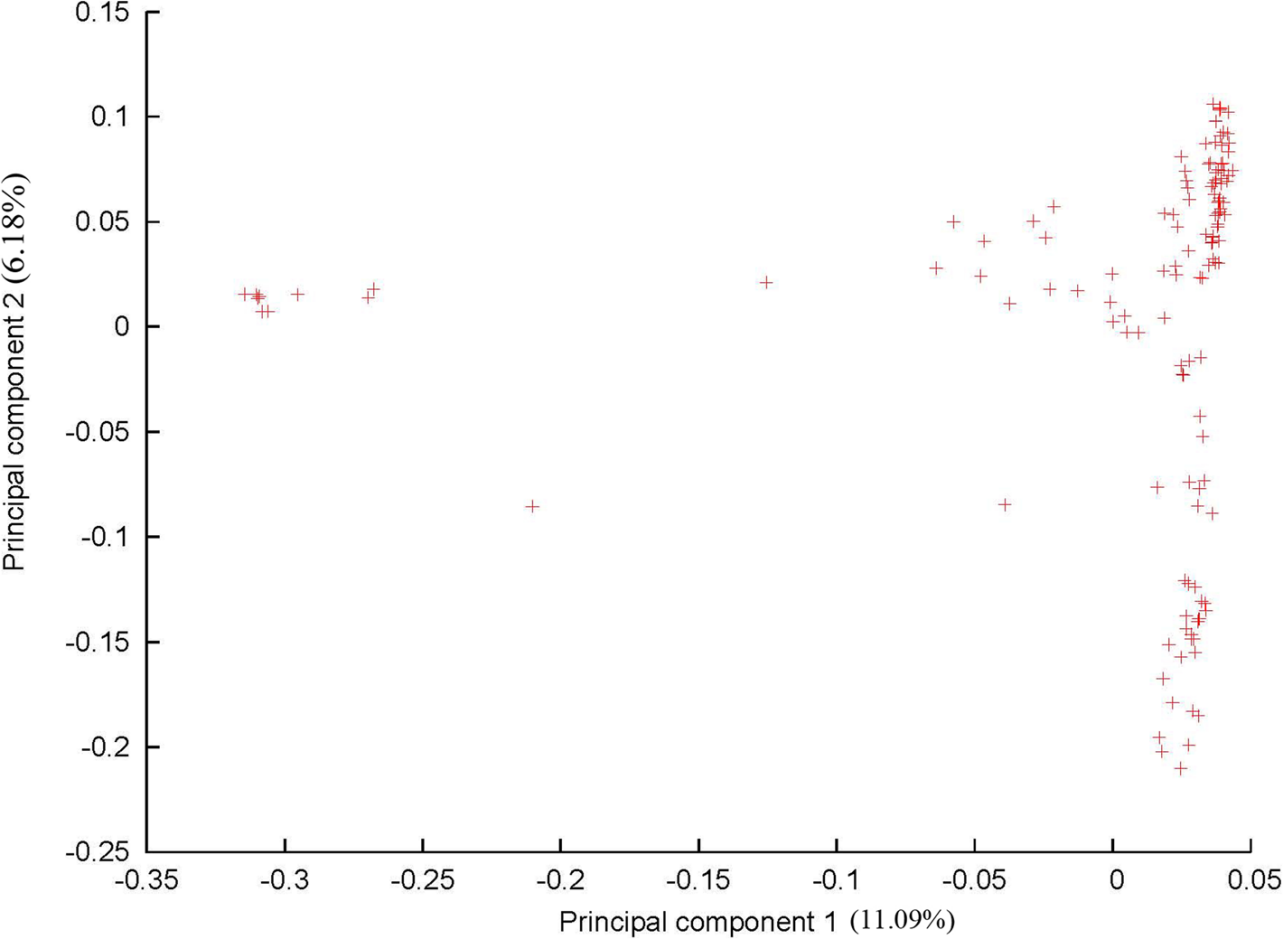
Principal component analysis on 3.8 million SNPs of Ting’s core collection. PC 1 and PC 2 refer to the first and second principal components, respectively. The numbers in parentheses refer to the proportion of variance explained by the corresponding axes. Symbols represent each variety in Ting’s core collection

**Fig. 3:**
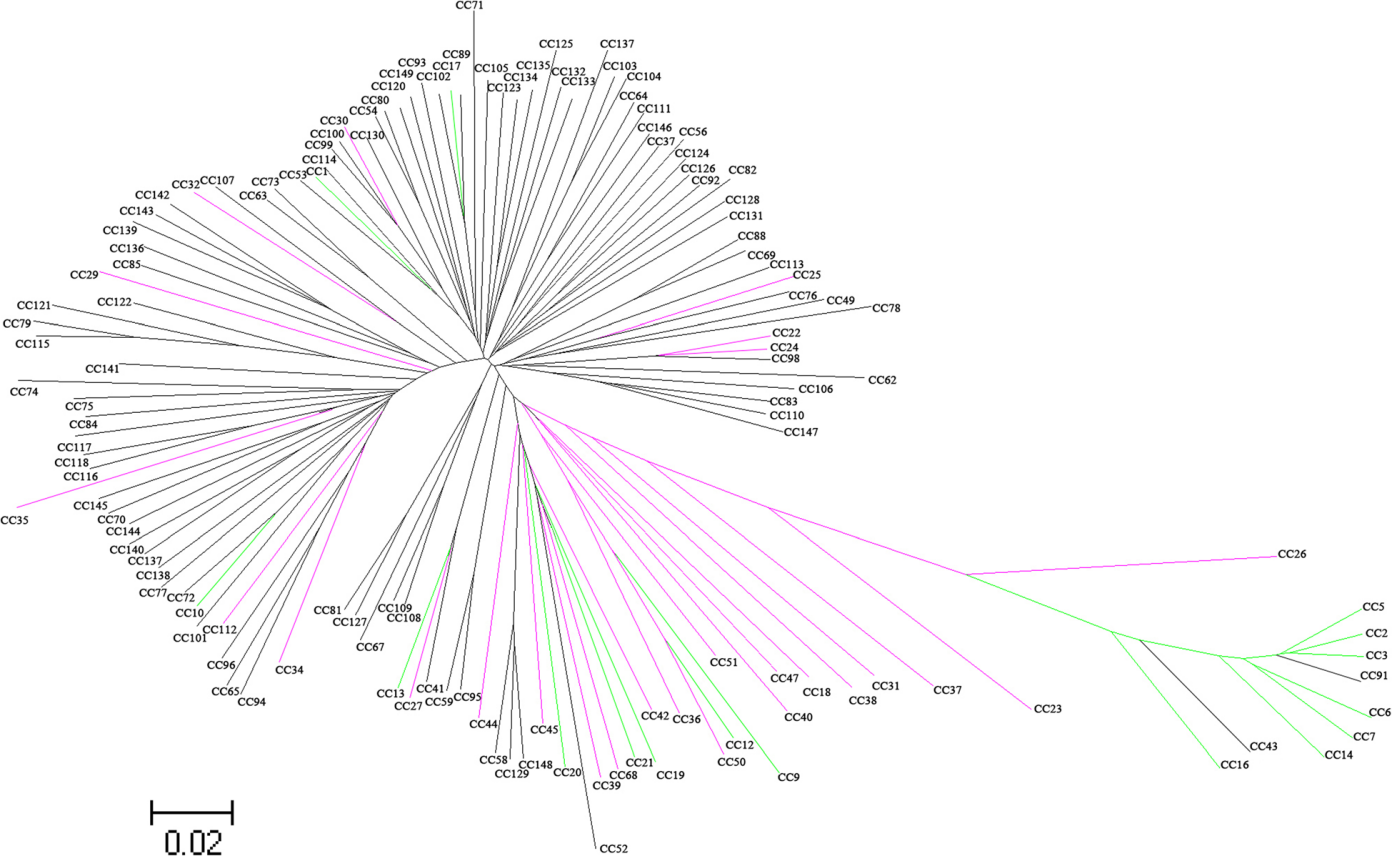
Unrooted neighbor-joining trees of 150 rice varieties in Ting’s core collection. Root with different colors represent the subpopulation identified in our previous study in which population structure was estimated by using 274 SSR markers (Zhang et al.*,* 2011), i.e. Black, green and purple represent *indica*, *japonica* and mixed, respectively

We performed PCA to identify the population structure of Ting’s core collection with all SNPs data, and we observed two subpopulations in Ting’s core collection (Fig. 2). The discrimination obtained via a NJ tree based on the SNP data was not identical to that based on Cheng’s index method (Additional file 2: Table S1) [19] and showed fairly consistent results with that from the PCA (Fig. 3). Moreover, the LD dropped to the half of its maximum value at a distance of 100~350 kb on the 12 chromosomes, which is agreement with previous measurements [5, 9, 22, 23] (Additional file 1: Figure S2).

### Relative kinship among varieties in Ting’s core collection and the effect of controlling type I error using EMMAX

In Ting’s core collection, most kinship estimates between varieties were zero, and none of the kinship values were larger than 0.5, indicating that these varieties were unrelated (Additional file 1: Figure S3).

Observed versus expected *P* values for each signal were graphed for estimating the effect of controlling for type I errors. As deviations from expected values demonstrate that the statistical analysis may cause spurious associations [24]. Our result indicated that the false positives were unlikely for all traits except grain length/width for the EMMAX method used in this study (Additional file 1: Figure S4).

### GWAS of 12 agronomic traits

**Fig. 4:**
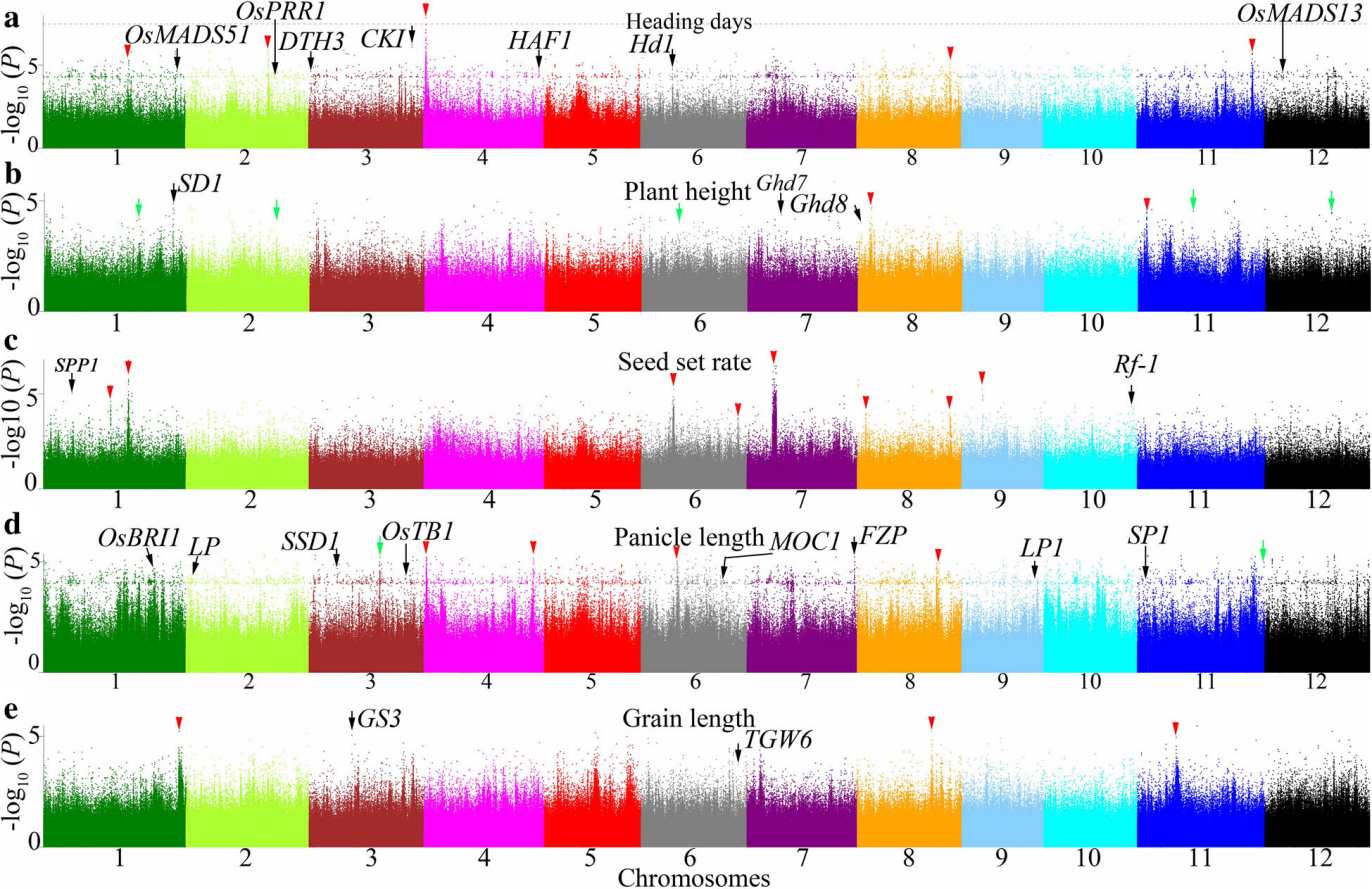
Manhattan plots of EMMAX for 5 agronomic traits in genome-wide association studies. Negative log10(*P*) values from a genome-wide scan are plotted against position on each of 12 chromosomes. **a** Manhattan plots of EMMAX for heading days. Red horizontal dashed line indicates the genome-wide significant threshold; **b** Manhattan plots of EMMAX for plant height; **c** Manhattan plots of EMMAX for seed set rate; **d** Manhattan plots of EMMAX for panicle length; **e** Manhattan plots of EMMAX for grain length. Black, red and green arrow represent the loci close to previous genes, new loci and identical in Guangzhou and Hangzhou, respectively

A total of 3,808,730 SNPs were included in a GWAS of 12 agronomic traits using the EMMAX method. Only one association signal’s -log_10_(*P*) value was higher than 6.58 (this value was the significant threshold in this study, please see methods section)—a signal for heading days (Fig. 4a). Thus, we used -log_10_(mBF) = 4.97 as the significance threshold for different traits in our study. A total of 1308 and 4272 significant loci were identified for the 12 agronomic traits in Guangzhou and Hangzhou, respectively (Table 1). The top-ranking candidate gene-based association signals for each trait are shown in Additional file 3: Table S4.

**Table 1:** Summary of association mapping results for 12 agronomic traits using EMMAX

Trait	Number of significant loci		Identical genome region (IRGSP 1.0)	
	Guangzhou	Hangzhou	Position (Guangzhou)	Position (Hangzhou)
Heading days	18	174	–	–
Plant height	3	2	–	–
Seed set rate	943	78	Chr05_25567352	Chr05_25408291
			Chr06_8915912~Chr06_9551431	Chr06_9230285
			Chr07_8779751	Chr07_8438294~Chr07_8467097
Panicle length	1	129	–	–
Grain length	5	7	–	–
Grain width	2	54	–	–
Grain length/width	0	3772	–	–
100 grains weight	0	1	–	–
Flag leaf length	3	3	–	–
Flag leaf width	9	31	–	–
Flag leaf length/width	323	19	Chr10_12103594~Chr10_12266458	Chr10_12442627
			Chr12_8018851~Chr12_8206256	Chr12_8234635
Panicle number per plant	1	2	–	–
Total	1308	4272	–	–

**Fig. 5:**
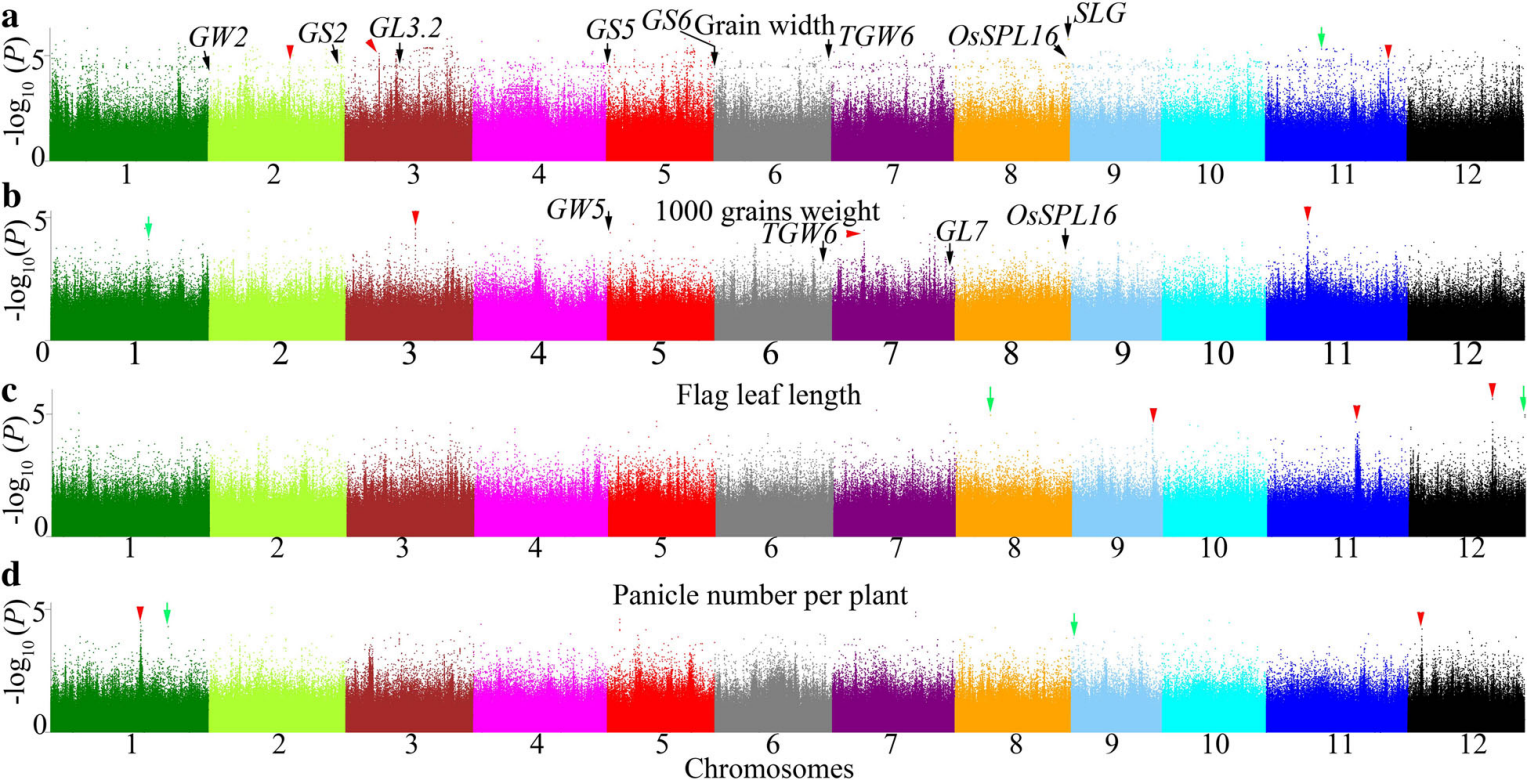
Manhattan plots of EMMAX for 4 agronomic traits in genome-wide association studies. Negative log10(*P*) values from a genome-wide scan are plotted against position on each of 12 chromosomes. **a** Manhattan plots of EMMAX for grain width; **b** Manhattan plots of EMMAX for 100 grains weight; **c** Manhattan plots of EMMAX for flag leaf length; **d** Manhattan plots of EMMAX for panicle number per plant. Black, red and green arrow represent the loci close to previous genes, new loci and identical in Guangzhou and Hangzhou, respectively

**Fig. 6:**
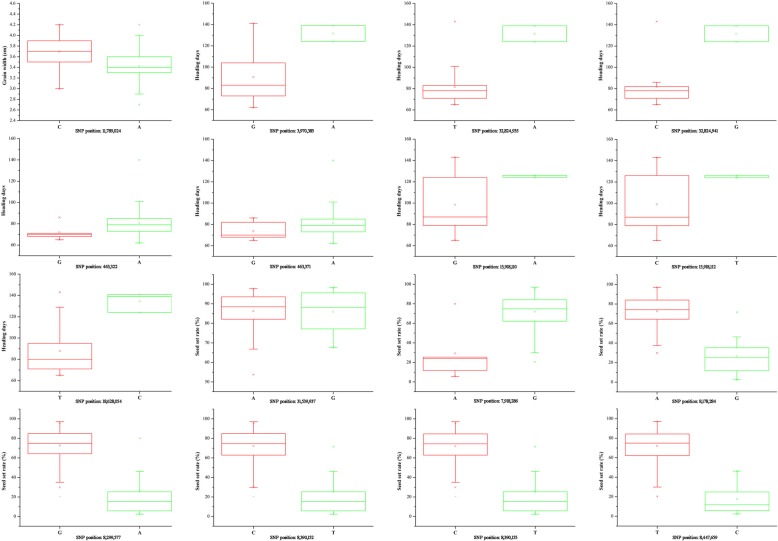
The box plots showing phenotypic distribution for Ting’s core collection carrying the different alleles at the top 16 significant SNPs in Table 3. The middle line indicates the median, the box indicates the range of the 25th to 75th percentiles of the total data, the whiskers indicate the inter-quartile range and the outer dots are outliers

Furthermore, Si et al. (2016) indicated that they considered analyzing the 11 predicted genes within the 260-kb interval centered on the index SNP from the GWAS given the estimated LD decay rate of about 100 to 200 kb [25]. Thus, we analyzed whether some of the significant detections for each trait were identical in the two locations according to the estimated distance of LD decay of 100 to 350 kb on the 12 chromosomes (Additional file 1: Figure S2). Three significant regions (located on chromosomes 5, 6 and 7) for seed set rate were detected both in Guangzhou and Hangzhou. Moreover, two significant regions for flag leaf length/width were detected (located on chromosomes 10 and 12) in both locations (Figs. 4b, d, 5a, b, c, d and Table 1). Moreover, we chose the top 16 most significant signals (*P* value < 1 × 10^− 6^) for in-depth analysis (Tables 2 and 3). The significant association signals with smaller *P* values and higher consecutive peaks for each trait are summarized in Table 3, Figs. 4 and 5, these signals might be located in candidate genes/regions. In addition, a detailed distribution of these new gene-based association signals is included in Additional file 4: Table S5 To confirm the effect of different alleles at the top 16 significant SNPs in the present study, we performed allelic analysis to these SNPs. Accessions in Ting’s core collection carrying different alleles for most of the 16 SNPs showed distinct discrepancies of phenotypes (Fig. 6).

**Table 2:** Information of new genome-wide significant association signals using EMMAX

Trait	Chromosome	SNP position (IRGSP 1.0)
Heading days	1	23,170,046~23,178,871
	2	23,561,650~23,647,315
	4	501,174~599,922
	8	24,711,213~24,788,877
	11	25,420,422~25,527,993
Plant height	8	4,006,947~4,099,049
	11	2,700,225~2,766,157
Seed set rate	1	18,520,148~18,579,781
	1	22,939,166~22,978,931
	6	8,983,019~9,267,052
	6	27,910,112~27,985,250
	7	7,750,765~9,020,981
	8	2,701,887~2,793,377
	8	23,506,423~23,515,115
	9	5,476,906~5,579,147
Panicle length	4	431,949~574,020
	4	30,763,510~31,085,620
	6	10,410,902~10,540,457
	8	19,760,055~19,773,993
Grain length	1	40,952,595~41,000,011
	8	18,807,278~18,877,159
	11	10,147,830~10,203,596
Grain width	3	11,852,705~11,908,957
	11	24,258,724~24,290,332
100 grains weight	3	20,308,101~20,374,967
	7	8,265,240~8,330,871
	11	9,821,720~9,835,992
Flag leaf length	9	19,484,679~19,503,424
	11	17,945,459~17,974,773
	12	17,992,241~18,042,367
Panicle number per plant	1	22,182,883~22,220,448
	12	4,146,868~4,199,211

**Table 3:** Top highest genome-wide significant association signals of agronomic traits using EMMAX

Trait	Chr.	Position (IRGSP 1.0)	Reference allele	Alternative allele	Alternative allele frequency	-log_10_(*P*)	*R*^*2*^ (%)^a^	Candidate/known gene^b^
Grain width	1	11,789,024	C	A	0.77	6.29	9.41	
Heading days	2	3,970,385	G	A	0.05	6.20	11.54	
	3	32,824,935	T	A	0.05	6.21	8.57	*CK1* [29]
	3	32,824,941	C	G	0.05	6.08	10.75	*CK1* [29]
	4	463,322	G	A	0.95	7.86	12.86	
	4	463,371	G	A	0.94	7.41	10.97	
	8	15,918,110	G	A	0.05	6.21	7.42	
	8	15,918,112	C	T	0.05	6.10	4.38	
	9	18,628,054	T	C	0.13	6.04	6.87	
Seed set rate	4	31,539,937	A	G	0.18	7.35	9.45	LOC_Os04g52940.1
	7	7,918,286	G	A	0.07	6.16	5.21	
	7	8,178,284	A	G	0.12	6.47	3.15	
	7	8,299,577	G	A	0.07	6.33	14.26	
	7	8,390,152	C	T	0.07	6.08	5.27	
	7	8,390,155	C	T	0.07	6.09	8.25	
	7	8,447,659	T	C	0.09	6.45	9.64	LOC_Os07g14800.1

Note: ^a^*R*^*2*^ represents the genetic variants explained by the significant SNPs. ^b^Gene ID of MSU rice genome annotation project (http://rice.plantbiology.msu.edu/)

In our study, we also identified some genes that were reported in previous studies according to the estimated distance of LD decay of 100 to 350 kb on the 12 chromosomes. We think a SNP is close to a cloned gene when it locates in 350 kb from the cloned gene. For heading days, significant association signals close to *OsMADS51* on chromosome 1, *OsPRR1* [26] on chromosome 2, *DTH3* [27] on chromosome 3, *CKI* [28] on chromosome 3, *HAF1* [29] on chromosome 4, *Hd1* [30] on chromosome 6 and *OsMADS13* [31] on chromosome 12 were detected (Fig. 4a and Table 4). For plant height, significant association signals close to *SD1* [32], *Ghd7* [33] and *Ghd8* [34] were identified (Fig. 4b and Table 4). For seed set rate, signals close to *SPP1* [35] and *Rf-1* [36] were found (Fig. 4c and Table 4). For panicle length, significant association signals close to *OsBRI1* [37], *LP* [38], *SSD1* [39], *FZP* [40], *LP1* [41] and *SP1* [42] were found (Fig. 4d and Table 4). For grain length, significant association signals close to *GS3* [43] and *TGW6* [44] were detected (Fig. 4e and Table 4). For grain width, significant association signals close to *GW2* [45], *GS2* [46], *GL3.2* [47], *GS5* [48], *GS6* [49], *TGW6* [44], *OsSPL16-GW8* [50] and *SLG* [51] were detected (Fig. 5a and Table 4). For 100 grains weight, significant association signals close to *GW5* [52], *TGW6* [43], *GL7* [53] and *OsSPL16* [50] were identified (Fig. 5b and Table 4).

**Table 4:** Top highest genome-wide significant association signals of agronomic traits using EMMAX

Trait	Significant loci	*-log*_*10*_ *(P)*	genes	Genes position
Heading days	Chr1_40,209,752	4.56	*OsMADS51*	40,344,329~40,364,584
	Chr2_24,800,400	4.42	*OSPRR1*	24,569,294~24,572,560
	Chr3_1,342,491	4.59	*DTH3*	1,269,856~1,271,783
	Chr3_32,824,935	6.21	*CKI*	32,999,502~33,006,898
	Chr4_32,895,071	4.69	*HAF1*	33,022,716~33,028,387
	Chr6_9,156,215	4.78	*Hd1*	9,336,359~9,338,643
	Chr12_5,600,578	4.55	*OsMADS13*	5,586,131~5,590,285
Plant height	Chr1_38,483,533	4.85	*SD1*	38,382,382~38,385,504
	Chr7_9,235,801	4.38	*Ghd7*	9,152,402~9,155,185
	Chr8_4,056,392	4.70	*Ghd8*	4,333,717~4,335,434
Seed set rate	Chr1_6,369,510	5.15	*SPP1*	6,528,797~6,630,463
	Chr10_18,962,735	4.41	*Rf-1*	18,935,690~18,942,573
Panicle length	Chr1_29,750,254	4.39	*OsBRI1*	29,927,543~29,931,487
	Chr2_9,035,894	4.31	*LP*	9,042,076~9,046,141
	Chr3_10,779,794	4.69	*SSD1*	10,684,315~10,688,955
	Chr7_28,613,922	5.20	*FZP*	28,299,591~28,301,089
	Chr9_16,891,286	4.12	*LP1*	17,182,867~17,188,378
	Chr11_7,007,154	4.23	*SP1*	7,193,230~7,198,552
Grain length	Chr3_16,876,884	4.12	*GS3*	16,729,501~16,735,109
	Chr6_25,249,340	4.46	*TGW6*	25,093,242~25,094,294
Grain width	Chr2_8,073,466	4.28	*GW2*	8,114,961~8,121,925
	Chr2_28,875,239	4.33	*GS2*	28,863,173~28,866,997
	Chr3_17,360,192	4.45	*GL3.2*	17,340,415~17,342,284
	Chr5_3,576,630	4.45	*GS5*	3,439,259~3,443,769
	Chr6_1,281,784	5.07	*GS6*	1,465,499~1,468,600
	Chr6_25,355,332	4.62	*TGW6*	25,093,242~25,094,294
	Chr8_26,162,707	4.61	*OsSPL16*	26,501,167~26,506,218
	Chr8_28,114,414	4.44	*SLG*	28,162,970~28,165,431
100 grains weight	Chr5_5,539,341	3.73	*GW5*	5,365,122~5,366,701
	Chr6_25,216,303	4.01	*TGW6*	25,093,242~25,094,294
	Chr7_24,377,379	3,69	*GL7*	24,664,168~24,669,324
	Chr8_26,475,471	3.78	*OsSPL16*	26,501,167~26,506,218

## Discussion

The abundant genetic variation in Ting’s core collection makes it an important reservoir of genetic diversity and potential source of beneficial alleles for rice breeding (Fig. 1). It is very difficult to mine and utilize the exotic genes in all the rice accessions (i.e., 775,000) in the world [54] by either linkage mapping or association mapping. The maximum population size used for GWAS was 1495 rice accessions in a previous study [10]. One of the methods of utilizing a large set of germplasm in a GWAS is to construct a core collection [16]. A rice core collection consisting of 150 accessions selected based on 48 morphological traits from 2262 accessions of Ting’s collection has been constructed and used in rice association mapping with low resolution [19, 20]. Therefore, we performed a GWAS by whole-genome re-sequencing for getting higher resolution within Ting’s core collection.

Although the population size of Ting’s core collection is smaller than that of three other populations [5, 8, 9], the phenotypic diversity of several agronomic traits was comparable to that of these populations or even higher for some agronomic traits. Moreover, more than 3.8 million SNPs in Ting’s core collection were developed. The ratio of SNPs to population size in Ting’s core collection is higher than that in previous studies in which the ratio were approximately 3.6 million SNPs to 517 rice landraces [5], 0.04 million SNPs to 413 diverse landraces and cultivars [9], 4.1 million SNPs to 950 worldwide varieties [6], 1.6 million SNPs to 1495 elite hybrid varieties [10] and 0.04 million SNPs to 176 *japonica* varieties [8]. Furthermore, a simpler population structure (Figs. 2 and 3), more rapid LD decay (Additional file 1: Figure S2) and more distant relatedness (Additional file 1: Figure S3) among accessions were found in Ting’s core collection than in other collections. The above mentioned information illuminates and supports the fact that Ting’s core collection is suitable for GWASs.

Population structure in the present study was not identical to that in our previous study [55, 56]. This discrepancy might be due to molecular markers density used in two studies. In our previous study, 274 SSR markers were included to detect the population structure while about 3.8 million SNPs were used in present study.

A total of 3,808,730 SNPs from 150 varieties were used for the GWAS (Additional file 2: Table S3). A mixed model was performed using EMMAX software [55, 56]. EMMAX not only can correct for a wide range of sample structures by explicitly accounting for pairwise relatedness between individuals, using high-density markers to model the phenotype distribution. But also can reduce computational time [55, 56]. The value obtained from a rough Bonferroni correction of *P* = 1/n, where n is the total number of markers used in the GWAS, is widely applied as the threshold *P* value for significance [5–8, 10]. The threshold *P* value for significance in our study was *P* ≤ 2.63 × 10^− 7^, corresponding to -log_10_(*P*) = 6.58. However, only one peak, i.e., one on chromosome 4 for heading days was higher than this threshold value in Fig. 4a. Hence, we chose a lower -log_10_(mBF) value as the significance threshold for different traits in our study (Table 1) because there will be no significant locus according to the theoretical threshold *P* value. We speculated that this result might due to population size in our study. However, Ting’s core collection is suitable for GWASs because the peaks located in well-known genes such as *SD1*, *GS2*, *GS3*, *GS5*, *GL7*, *GW8* and *TGW6* were also much lower than the theoretical threshold value (Figs. 4 and 5).

In our study, some significant association signals were identified through a GWAS of Ting’s core collection. First, loci significantly associated with agronomic traits were uncovered close to cloned genes such as *Hd1*, *SD1*, *Ghd7*, *GW8*, and *GL7* (Figs. 4, 5 and Table 4) that were reported in previous studies. Moreover, some of these loci were located by coincidence in these genes, and they might be natural variations of these genes, which could be functional (Table 2 and Additional file 3: Table S4). Second, Si et al. [25] indicated that some significant loci within the distance of LD decay might be identical to each other. However, there were no identical significant loci in the two locations overall (Table 1), but some identical significant regions were discovered in the two locations when the estimated distance of LD decay of 100 to 350 kb was considered in Ting’s core collection (Table 1, Figs. 4 and 5). Third, some new significant association signals that might be candidate genes were detected in our study (Figs. 4, 5 and Additional file 4: Table S5). Some peaks of these candidate genes such as the peak on chromosome 4 for heading days (Fig. 4a) were even higher than the threshold value. Further, the peak on chromosome 11 for heading days (Fig. 4a) was higher than that of some famous genes such as *Hd1*. It would be valuable to test the functions of these candidate genes because some loci or regions were also detected by previous studies. For instance, the region on chromosome 8 for plant height, the region at position 23,300,000 on chromosome 1 for heading days and the region at position 21,650,000 on chromosome 2 were found to be significantly associated with related traits in the study of Zhao et al. [9].

## Conclusions

In this study, Ting’s core collection showed abundant genetic variation for agronomic traits and was proved to be a suitable natural population that could be comparable to other populations used in previous GWASs. Moreover, according to this study, core collections constructed from large natural populations of other plants might be good choices for GWASs. Furthermore, some natural variations in cloned genes were founded in this study, and these variations could be used for functional analysis of these genes. In addition, new candidate genes identified in this study could be very useful for rice improvement. In sum, this study provided important information for further mining these elite genes within Ting’s core collection and using them for rice breeding.

## Methods

### Plant material

Ting’s core collection with 150 accessions of rice landraces [18], was used in this study. The information for these accessions is shown in Additional file 2: Table S1.

### Phenotyping

In total, 12 agronomic traits of Ting’s core collection were measured in two locations. The methods of measuring these 12 agronomic traits were identical to those described in detail in our previous study [20].

A randomized complete block design with three replications was used in two locations. First, Ting’s core collection was cultivated at the farm of South China Agricultural University, Guangzhou (23°16′’ N, 113°8′ E), during the late season (July–November) in 2009. The design and methods of this research in Guangzhou were described in detail in our previous study [20]. Second, Ting’s core collection was cultivated at the farm of China National Rice Research Institute, Hangzhou (30°3′ N, 120°2′ E), during the late season (May–October) in 2016. A randomized complete block design with three replications, as in Guangzhou, was used during this season in Hangzhou. The space between rows and between plants was set to 26 and 20 cm, respectively. Twenty-four plants of each variety were grown in four rows with 6 plants per row. For each block, the five plants in the middle position of the second and third row of each variety were selected to prevent edge effects. The broad-sense heritability (*H*^*2*^) was calculated as ${H}^2={\sigma}_{\mathrm{g}}^2/\left({\sigma}_{\mathrm{g}}^2+{\sigma}_{\mathrm{e}}^2\right)$, where ${\sigma}_{\mathrm{g}}^2$ is the genetic variance, ${\sigma}_{\mathrm{e}}^2$ is the environmental variance.

### DNA isolation and genome sequencing

Total genomic DNA was extracted using a modified SDS method. Then, each landrace’s DNA was sheared randomly into ~ 500-bp fragments by Covaris, and the DNA fragments were loaded on 2% agarose gels. Fragments of ~ 500 bp were recovered and purified, and adapters were then added to each fragment. After making libraries for the clusters, they were loaded into an Illumina HiSeq™ 4000 for 2× 150-bp paired-end sequencing at 6~7-fold genome coverage.

The 150-bp paired-end reads were mapped onto the rice reference genome (IRGSP 1.0) using bwamem with the –M option in BWA software [57]. The mapped reads were realigned by using RealignerTargetCreator and IndelRealigner in GATK [58]. UnifiedGenotyper in GATK was used with the −glm BOTH option to label SNPs and indels. After removing nucleotide variants with a missing rate ≥ 0.25 and a minor allele frequency > 0.05, a total of 3,808,730 SNPs and 391,756 indels were generated.

### Population genetic analyses

Principal component analysis (PCA), construction of a neighbor-joining (NJ) tree, determination of LD decay level and kinship analysis among landraces were performed based on SNPs. The population structure of the 150 varieties was estimated with PCA by using the software EIGENSTRAT [59]. PHYLIP version 3.695 software (http://evolution.genetics.washington.edu/phylip/getme-new1.html) was used to construct the NJ tree on the basis of similarity measures. The software MEGA V5.2 was used to observe the NJ tree [60]. The LD in Ting’s core collection was evaluated using squared Pearson’s correlation coefficients (*r*^*2*^) calculated with the −r2 command in the software PLINK [61]. A Q matrix was obtained from the membership probability of each variety using ADMIXTURE Version 1.22 software [62]. The Q matrix was used for further association mapping. The Loiselle algorithm was chosen to construct a kinship matrix (K) with the software SPAGeDi [63]. Moreover, all negative kinship values were set to zero.

### GWAS

A total of 3,808,730 SNPs from 150 varieties were used for GWAS. A mixed model was performed using EMMAX software [56]. *P* ≤ 2.63 × 10^− 7^ (*P* = 1/n, *n* = total number of markers used [7], which is a rough Bonferroni correction, corresponding to -log_10_(*P*) = 6.58). However, no significant loci were detected based on this threshold, hence, we calculated another significance threshold, i.e., a minimum Bayes factor (mBF), based on the *P* value threshold for significance. The mBF was calculated using the following formula: mBF = −e_*_P_*_ln(*P*) [64]. Thus, the significance threshold in this study was -log_10_(*P*) = 4.97.

## Additional files

Additional file 1: **Figure S1.** SNP distribution along position in each chromosome. **Figure S2.** Genome-wide average LD decay estimated in Ting’s core collection on 12 chromosomes. **Figure S3.** Distribution of pairwise relative 1 kinship values based on 3.8 million SNPs in Ting’s core collection. The height of blue bar represents the percentage of varieties in different range of kinships. **Figure S4.** Plots of observed versus expected *P*-values using EMMAX for 12 agronomic traits. Red symbol represents expected *P*-values, and Blue symbol represents observed *P*-values. (DOCX 1084 kb)

Additional file 2: **Table S1.** Accessions, variety names, origin and germplasm types of 150 rice varieties in Ting’s core collection. **Table S2.** Re-sequencing average read depth and coverage in Ting’s core collection. **Table S3.** Summary of categorized SNPs and InDels. (DOC 246 kb)

Additional file 3: **Table S4.** List of all *P*-value ranked genes in the gene-based association analysis of heading days/plant height/seed set rate/panicle length/grain length/100 grains weight/flag leaf length/flag leaf width/panicle number per plant. (XLSX 169 kb)

Additional file 4: **Table S5.** List of new loci in association analysis of heading days/plant height/seed set rate/panicle length/grain length/grain width/100 grains weight/ flag leaf length/panicle number per plant. (XLSX 51 kb)

## Data Availability

The datasets used during the current study are available from the corresponding author on reasonable request.

## Abbreviations

CDS: Coding sequence
DNA: Deoxyribonucleic acid
EMMAX: Efficient mixed model association eXpedited
IRGSP: International Rice Genome Sequencing Project
QTL: Quantitative trait locus
SDS: Sodium dodecyl sulfate
SNPs: Single Nucleotide Polymorphisms
SSR: Simple Sequence Repeats
