# Supplementary material for: Genome-wide association study of important agronomic traits within a core collection of rice (Oryza sativa L.)
Source: BMC Plant Biol. 2019 Jun 17;19:259. doi: 10.1186/s12870-019-1842-7 (PMC6580581; doi:10.1186/s12870-019-1842-7)
Supplement: Supplementary file 1 — Figure S1. SNP distribution along position in each chromosome. Figure S2. Genome-wide average LD decay estimated in Ting’s core collection on 12 chromosomes. Figure S3. Distribution of pairwise relative 1 kinship values based on 3.8 million SNPs in Ting’s core collection. The height of blue bar represents the percentage of varieties in different range of kinships. Figure S4. Plots of observed versus expected P-values using EMMAX for 12 agronomic traits. Red symbol represents expected P-values, and Blue symbol represents observed P-values. (DOCX 1084 kb) [file 12870_2019_1842_MOESM1_ESM.docx]

**
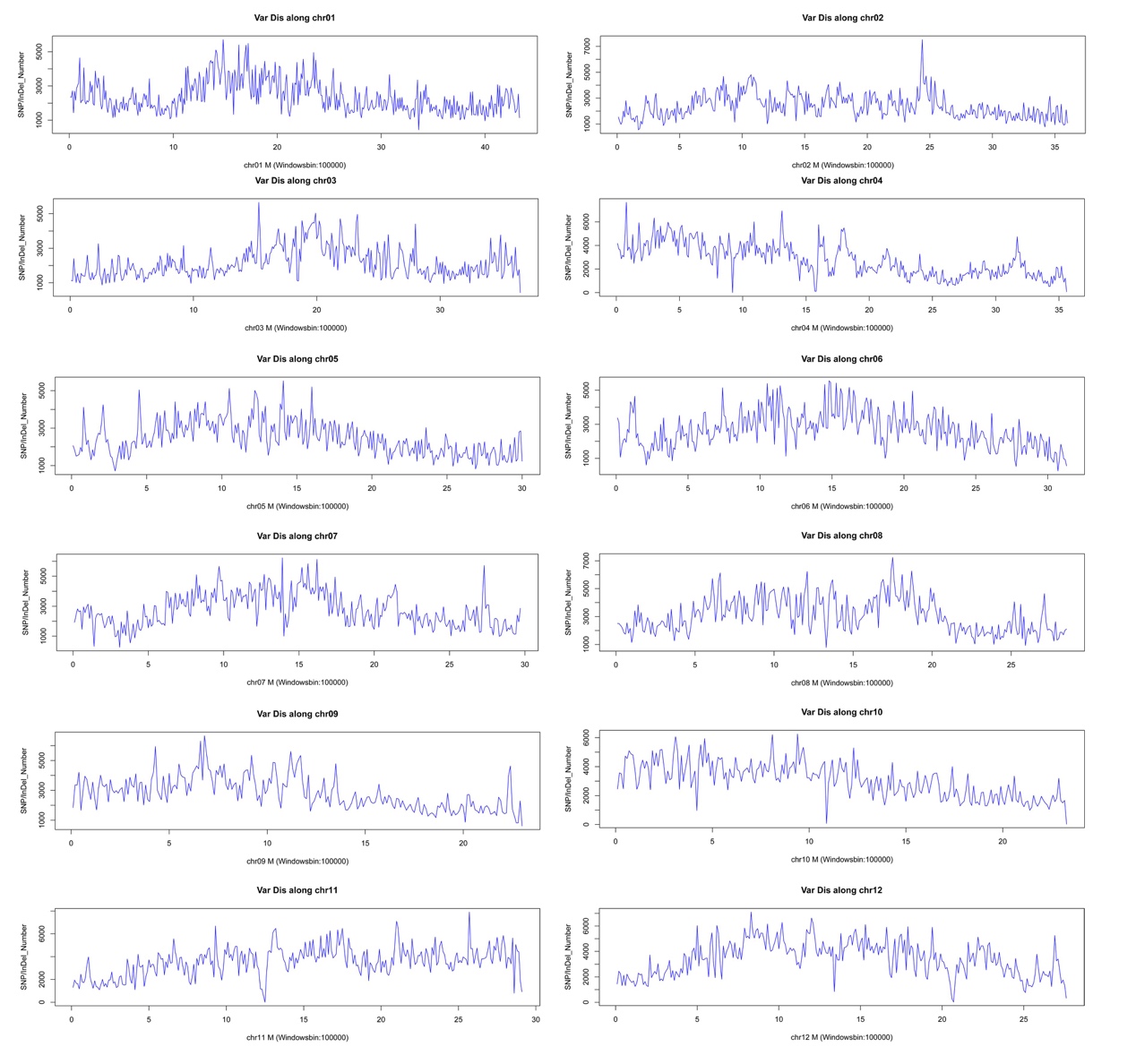
Fig. S1** SNP distribution along position in each chromosome.

**
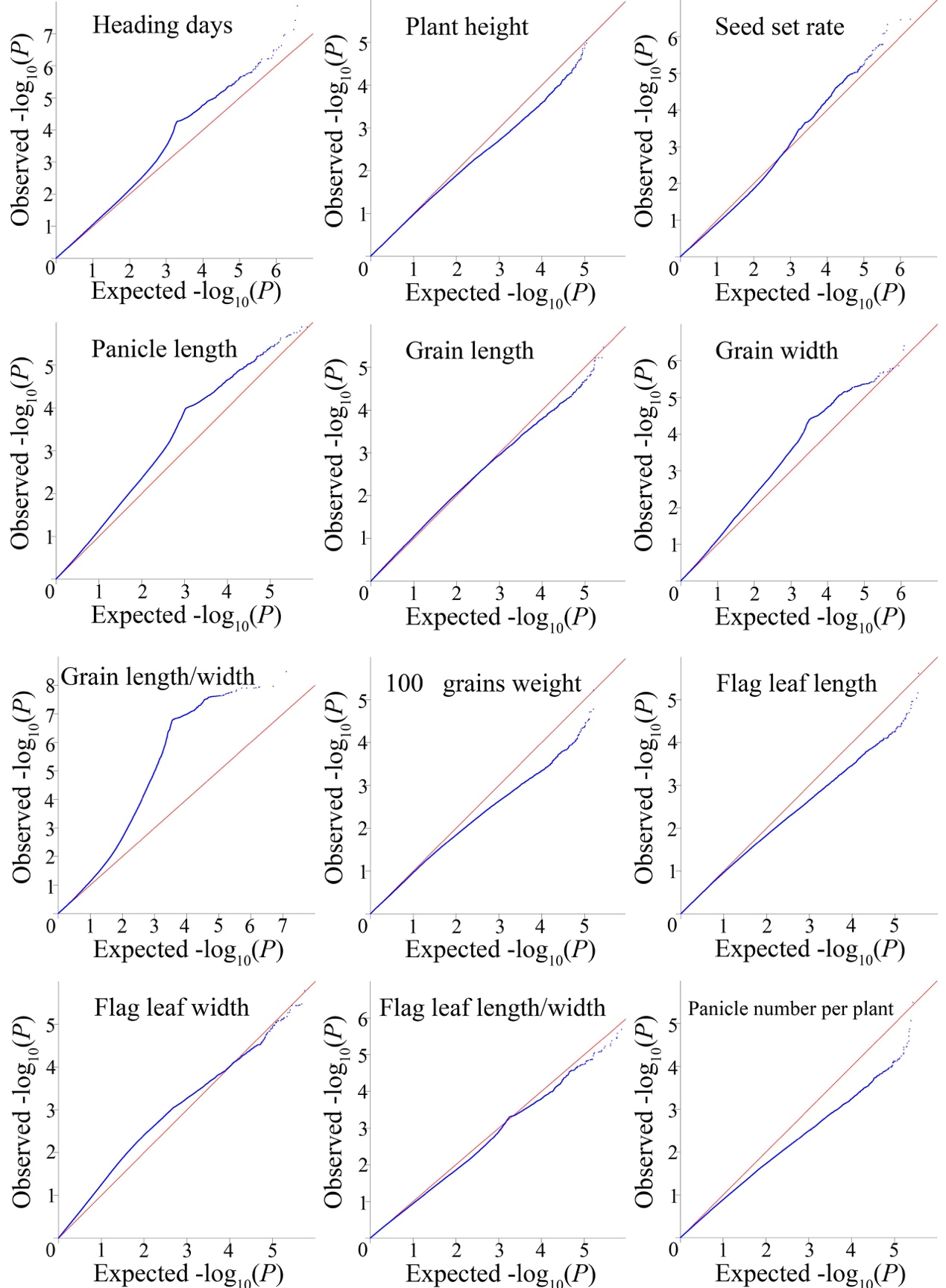
**

**Fig. S2** Genome-wide average LD decay estimated in Ting’s core collection on 12 chromosomes.

**
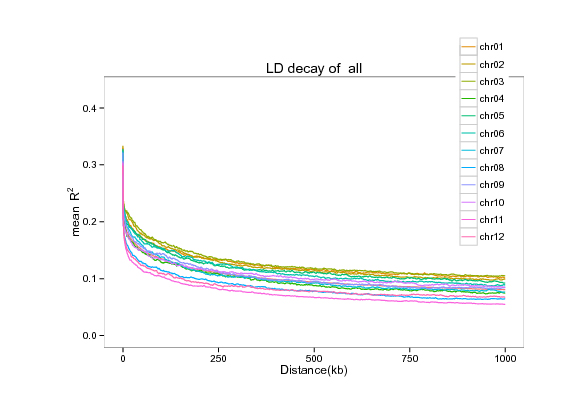
**

**Fig. S3** Distribution of pairwise relative 1 kinship values based on 3.8 million SNPs in Ting’s core collection. The height of blue bar represents the percentage of varieties in different range of kinships.

**

**

**Fig. S4** Plots of observed versus expected *P*-values using EMMAX for 12 agronomic traits. Red symbol represents expected *P*-values, and Blue symbol represents observed *P*-values.
