# Supplementary material for: Genome-wide association study of important agronomic traits within a core collection of rice (Oryza sativa L.)
Source: BMC Plant Biol. 2019 Jun 17;19:259. doi: 10.1186/s12870-019-1842-7 (PMC6580581; doi:10.1186/s12870-019-1842-7)
Supplement: Supplementary file 2 — Table S1. Accessions, variety names, origin and germplasm types of 150 rice varieties in Ting’s core collection. Table S2. Re-sequencing average read depth and coverage in Ting’s core collection. Table S3. Summary of categorized SNPs and InDels. (DOC 246 kb) [file 12870_2019_1842_MOESM2_ESM.doc]

**Table S1** Accessions, variety names, origin and germplasm types of 150 rice varieties in Ting’s core collection

| **Acc.** | **Variety names** | **Origin** | ***Indica* vs.**  ***Japonica***  ***(marker)*** | **Acc.** | **Variety names** | **Origin** | ***Indica* vs.**  ***Japonica***  ***(marker)*** |
| --- | --- | --- | --- | --- | --- | --- | --- |
| CC1 | Yin guang | Japan | *J* | CC76 | Guang ye hong mi | South China | *I* |
| CC2 | Ao guo 5-B | Japan | *J* | CC77 | Da nuo | South China | *I* |
| CC3 | Ai you | Japan | *J* | CC78 | Bai xu | South China | *I* |
| CC4 | Tie geng yi shi ao | Yangtze River region | *J* | CC79 | Mao he | South China | *I* |
| CC5 | Guo zhu | Japan | *J* | CC80 | Xu zai | South China | *I* |
| CC6 | Ben dao | North China | *J* | CC81 | Dong an hou zi pu xiao he | Central China | *I* |
| CC7 | Mang shui dao | Yangtze River region | *J* | CC82 | Tie gu pao | Central China | *I* |
| CC8 | Bai mang gao li han dao bai | North China | *J* | CC83 | Chi mao zhan | South China | *I* |
| CC9 | Jiu yue han | Northeast China | *J* | CC84 | Hu bei zao | Central China | *I* |
| CC10 | Bi jie ma wei hong gu | Yunnan-Kweichow Plateau | *J* | CC85 | Ya jing mi | South China | *I* |
| CC11 | Ai da tou | Yangtze River region | *J* | CC86 | Ba shi zi | Central China | *I* |
| CC12 | Gui zao bai he | Yangtze River region | *J* | CC87 | Dong jun zi | Central China | *I* |
| CC13 | Xiang dao | North China | *J* | CC88 | Early | Unknown | *I* |
| CC14 | Zi jin gu | Northeast China | *J* | CC89 | Nuo | South China | *I* |
| CC15 | Xiang chuan | Japan | *J* | CC90 | Gui zhao he 2 | Japan | *I* |
| CC16 | Nagabo | Taiwan | *J* | CC91 | Hei nuo | Unknown | *I* |
| CC17 | Bai ke da nuo | South China | *J* | CC92 | Da yi mao | Central China | *I* |
| CC18 | San pai zhong | South China | *J* | CC93 | Gai cao zhan | Central China | *I* |
| CC19 | Kai xuan | Japan | *J* | CC94 | Gamal | Unknown | *I* |
| CC20 | Shi ban zhan | North China | *J* | CC95 | Bu gou wei | South China | *I* |
| CC21 | Hei ke da nuo | South China | *J* | CC96 | Bai ke xi nuo | South China | *I* |
| CC22 | Shen shui wan dao | Yangtze River region | AD | CC97 | Ben dao | North China | *I* |
| CC23 | Hong ben dao | Yangtze River region | AD | CC98 | Ba xian shu | Japan | *I* |
| CC24 | Duan mang zi jin gu | Northeast China | AD | CC99 | Guang hong mi dao | Yangtze River region | *I* |
| CC25 | Bei jing jiang mi | North China | AD | CC100 | Wu mang yan guo qing | North China | *I* |
| CC26 | Daeri | Celebes | AD | CC101 | Chang xu nuo | South China | *I* |
| CC27 | Jian tou nuo | South China | AD | CC102 | Jiang wan 15 | Central China | *I* |
| CC28 | Long you man dao | Yangtze River region | AD | CC103 | Bai hua er | South China | *I* |
| CC29 | Kun shan zhu zhou dao | Yangtze River region | AD | CC104 | Liu chang xian | South China | *I* |
| CC30 | Huang ke zao 2 | Yangtze River region | AD | CC105 | Bai yin 3 | South China | *I* |
| CC31 | Sheng fang da bai gu | North China | AD | CC106 | Shui zao huang pi | South China | *I* |
| CC32 | Xiao dou | Japan | AD | CC107 | Yin 2 dong 7 | South China | *I* |
| CC33 | Poetih | Celebes | AD | CC108 | Hou ma | South China | *I* |
| CC34 | Tebaro | Sumbawa | AD | CC109 | Dong zhu 2 | South China | *I* |
| CC35 | Ao hua da gui tou hong | Yangtze River region | AD | CC110 | Hong gen da mi | South China | *I* |
| CC36 | Hui bei zi | Yunnan-Kweichow Plateau | AD | CC111 | Ben cheng guan yin zhan | Central China | *I* |
| CC37 | Ba shi zi | Yangtze River region | AD | CC112 | Xi miao gu | South China | *I* |
| CC38 | Zao sheng da ye | Japan | AD | CC113 | 186-zao guan yin zhan | Central China | *I* |
| CC39 | Bnlastog | Low latitude region | *I* | CC114 | Chang mang hei ma zao | Yunnan | *I* |
| CC40 | Nuo mi | North China | *I* | CC115 | Shui tian zhan gu nuo | South China | *I* |
| CC41 | Xi chuan huang liu | South China | *I* | CC116 | Chang han da hua ke | South China | *I* |
| CC42 | Hei ju dao | Yangtze River region | *I* | CC117 | Da he | South China | *I* |
| CC43 | Guang fuⅠ | Taiwan | *I* | CC118 | Zeng cheng hei nuo | South China | *I* |
| CC44 | Zhong qi jia qing | Yangtze River region | *I* | CC119 | Mao he | South China | *I* |
| CC45 | Ⅲ-49-4xi chuan huang | Taiwan | *I* | CC120 | Bai gu zhan | Central China | *I* |
| CC46 | Xin xian li | Yangtze River region | *I* | CC121 | Die zhi | South China | *I* |
| CC47 | Da liu tiao dao | Yangtze River region | *I* | CC122 | You zhan | South China | *I* |
| CC48 | Bai ke | South China | *I* | CC123 | Chang sha wu qu wan dao | Central China | *I* |
| CC49 | Chuan chi 1 | Central China | *I* | CC124 | Tong ling hu nan xian | Central China | *I* |
| CC50 | Tai nong 46 | Taiwan | *I* | CC125 | Xiao mao dao | Central China | *I* |
| CC51 | Ba chong sui | Japan | *I* | CC126 | Jing xian si qu er gan | Central China | *I* |
| CC52 | Yun nan bai | Central China | *I* | CC127 | Zi xing er qu si dou xu | Central China | *I* |
| CC53 | Liao yang ben di 4 | Northeast China | *I* | CC128 | Chen hui fu dao | Central China | *I* |
| CC54 | You zhan hong | South China | *I* | CC129 | Bai gan zi | Central China | *I* |
| CC55 | Hei nuo | South China | *I* | CC130 | Han lu wei zhan | Central China | *I* |
| CC56 | Xian zi zhan | Central China | *I* | CC131 | Xin hua san qu tang mao zhan | Central China | *I* |
| CC57 | Da tou meng | Central China | *I* | CC132 | Ta gu zhan | Central China | *I* |
| CC58 | Chi bai gan zhan | Central China | *I* | CC133 | Mian tiao zhan | Central China | *I* |
| CC59 | Zeng cheng xiang shan zhan | South China | *I* | CC134 | Hu guang zhan | Central China | *I* |
| CC60 | Cang wu shan he zhan | South China | *I* | CC135 | Jiang an da ye zao | Central China | *I* |
| CC61 | Da gu zao | South China | *I* | CC136 | Tie ban zhan | Central China | *I* |
| CC62 | Jie yang dong liao zhong | South China | *I* | CC137 | Da gu zao | Central China | *I* |
| CC63 | Nan xiong ku gua zao | South China | *I* | CC138 | Ding nan dong zhan | Central China | *I* |
| CC64 | Chang mang | South China | *I* | CC139 | Lao wu gu | Central China | *I* |
| CC65 | Mandi | Celebes | *I* | CC140 | Xin ban chang ke zi | Central China | *I* |
| CC66 | Bai gu | South China | *I* | CC141 | Bai zhan gu | Central China | *I* |
| CC67 | Hong zao gu | Yunnan-Kweichow Plateau | *I* | CC142 | Si chuan zhan | Central China | *I* |
| CC68 | Cang wu shan he zhan | South China | *I* | CC143 | Gao jiao gui hua | Central China | *I* |
| CC69 | Zao die zhan gu | Central China | *I* | CC144 | Chang shu wu wi dao | Central China | *I* |
| CC70 | Su zhou zhan | Central China | *I* | CC145 | Da nuo bai dong | Central China | *I* |
| CC71 | Yang zhan 3 | South China | *I* | CC146 | Lin chuan da ye zao | South China | *I* |
| CC72 | Luo ding zhan 1 | South China | *I* | CC147 | Da bai cao | North China | *I* |
| CC73 | Gen yin 29 | South China | *I* | CC148 | Chang ning wu qu nan tou zhan | Central China | *I* |
| CC74 | Wu ke nuo | South China | *I* | CC149 | Pi xian da ye zi | Central China | *I* |
| CC75 | Hua bai ke | South China | *I* | CC150 | Xi zi zhan | Central China | *I* |

Note: *Indica* or *japonica* characteristic were identified in our previous study [19], i.e. *I*-*indica*, *J*-*japonica*, and AD-admixed.

**Table S2** Re-sequencing average read depth and coverage in Ting’s core collection

| **Acc.** | **Average depth** | **Coverage (%)** |  | **Acc.** | **Average depth** | **Coverage (%)** |
| --- | --- | --- | --- | --- | --- | --- |
| CC1 | 5.06 | 80.50 |  | CC58 | 7.67 | 84.47 |
| CC2 | 6.09 | 90.96 |  | CC59 | 5.81 | 80.37 |
| CC3 | 7.19 | 91.70 |  | CC60 | 7.82 | 81.71 |
| CC5 | 9.56 | 91.86 |  | CC61 | 6.22 | 81.42 |
| CC6 | 7.79 | 89.78 |  | CC62 | 5.81 | 81.74 |
| CC7 | 9.22 | 89.95 |  | CC63 | 9.67 | 84.29 |
| CC9 | 8.78 | 85.17 |  | CC64 | 8.79 | 81.83 |
| CC12 | 5.94 | 82.28 |  | CC65 | 7.07 | 82.35 |
| CC13 | 6.67 | 80.93 |  | CC67 | 6.86 | 79.94 |
| CC14 | 8.57 | 89.28 |  | CC69 | 7.07 | 83.84 |
| CC16 | 8.74 | 91.82 |  | CC70 | 9.22 | 84.67 |
| CC17 | 6.78 | 82.81 |  | CC71 | 8.55 | 82.74 |
| CC18 | 7.25 | 83.29 |  | CC72 | 7.67 | 80.05 |
| CC19 | 3.50 | 76.63 |  | CC73 | 8.51 | 83.90 |
| CC20 | 11.72 | 84.15 |  | CC75 | 7.21 | 80.63 |
| CC21 | 6.37 | 81.79 |  | CC76 | 7.49 | 80.92 |
| CC22 | 8.40 | 84.03 |  | CC77 | 6.80 | 81.31 |
| CC23 | 8.05 | 86.16 |  | CC78 | 8.75 | 81.41 |
| CC24 | 7.15 | 81.53 |  | CC79 | 8.17 | 82.13 |
| CC25 | 6.03 | 81.32 |  | CC80 | 7.35 | 81.74 |
| CC26 | 6.51 | 86.39 |  | CC81 | 7.77 | 83.76 |
| CC27 | 6.26 | 79.74 |  | CC82 | 7.14 | 83.68 |
| CC29 | 9.61 | 83.79 |  | CC83 | 8.66 | 82.43 |
| CC30 | 8.63 | 83.16 |  | CC84 | 6.64 | 81.78 |
| CC31 | 6.41 | 84.60 |  | CC85 | 6.89 | 80.65 |
| CC32 | 5.31 | 80.16 |  | CC86 | 7.28 | 82.12 |
| CC33 | 6.00 | 81.59 |  | CC88 | 5.46 | 80.66 |
| CC35 | 6.74 | 84.68 |  | CC89 | 6.27 | 80.39 |
| CC36 | 7.92 | 84.20 |  | CC91 | 7.80 | 90.68 |
| CC37 | 8.79 | 90.04 |  | CC93 | 4.89 | 81.73 |
| CC38 | 8.41 | 84.99 |  | CC94 | 6.31 | 81.74 |
| CC39 | 6.24 | 82.56 |  | CC95 | 5.70 | 81.25 |
| CC40 | 6.82 | 84.71 |  | CC96 | 6.93 | 82.55 |
| CC41 | 8.62 | 82.51 |  | CC98 | 2.62 | 73.49 |
| CC42 | 7.33 | 82.47 |  | CC99 | 7.40 | 82.16 |
| CC43 | 8.20 | 91.53 |  | CC100 | 7.65 | 82.38 |
| CC44 | 4.80 | 79.87 |  | CC101 | 7.75 | 82.92 |
| CC45 | 5.79 | 81.20 |  | CC102 | 7.26 | 81.16 |
| CC47 | 7.30 | 82.47 |  | CC103 | 6.44 | 80.80 |
| CC48 | 7.71 | 82.73 |  | CC104 | 7.39 | 80.71 |
| CC49 | 7.39 | 81.53 |  | CC105 | 7.68 | 81.28 |
| CC50 | 4.60 | 81.72 |  | CC106 | 6.37 | 81.82 |
| CC51 | 7.13 | 81.80 |  | CC107 | 3.90 | 78.73 |
| CC52 | 10.68 | 89.30 |  | CC108 | 8.59 | 81.83 |
| CC53 | 6.72 | 81.39 |  | CC109 | 9.23 | 82.33 |
| CC54 | 5.83 | 79.81 |  | CC110 | 8.90 | 80.71 |
| CC56 | 6.40 | 83.77 |  | CC111 | 7.55 | 80.95 |
| CC112 | 7.40 | 80.37 |  | CC131 | 8.34 | 84.77 |
| CC113 | 5.68 | 79.93 |  | CC132 | 9.58 | 84.63 |
| CC114 | 7.06 | 81.09 |  | CC133 | 6.98 | 82.81 |
| CC115 | 6.20 | 80.79 |  | CC134 | 7.77 | 82.82 |
| CC116 | 7.41 | 81.47 |  | CC135 | 7.53 | 84.26 |
| CC117 | 7.74 | 81.77 |  | CC136 | 8.41 | 83.98 |
| CC118 | 8.08 | 81.89 |  | CC137 | 8.20 | 85.17 |
| CC119 | 8.53 | 82.36 |  | CC138 | 6.05 | 84.34 |
| CC120 | 6.87 | 80.72 |  | CC139 | 9.12 | 84.49 |
| CC121 | 5.91 | 80.42 |  | CC140 | 6.44 | 83.45 |
| CC122 | 9.08 | 84.58 |  | CC141 | 6.12 | 83.17 |
| CC123 | 7.43 | 81.39 |  | CC142 | 6.68 | 82.99 |
| CC124 | 8.45 | 81.96 |  | CC143 | 5.51 | 82.62 |
| CC125 | 8.01 | 82.45 |  | CC144 | 4.22 | 80.03 |
| CC126 | 5.42 | 81.47 |  | CC145 | 9.45 | 85.16 |
| CC127 | 7.52 | 82.71 |  | CC146 | 7.34 | 83.86 |
| CC128 | 5.08 | 82.22 |  | CC147 | 9.42 | 83.72 |
| CC129 | 8.64 | 84.10 |  | CC148 | 8.65 | 82.53 |
| CC130 | 6.48 | 82.56 |  | CC149 | 6.61 | 83.98 |

**Table S3** Summary of categorized SNPs and InDels

| **Location category** | **SNPs** | **InDels** |
| --- | --- | --- |
| promoter | 1,745,893 | 290,443 |
| three_prime_UTR | 223,704 | 41,103 |
| exon | 27,776 | 4,197 |
| CDS | 386,562 | 27,040 |
| five_prime_UTR | 126,732 | 28,464 |
| intron | 1,298,163 | 509 |
| Total | 3,808,730 | 391,756 |
